# A Case with Hepatic Involvement Mimicking Metastatic Disease in Multiple Myeloma

**DOI:** 10.1155/2020/5738319

**Published:** 2020-07-17

**Authors:** Mert Erciyestepe, Tarık Onur Tiryaki, İpek Yönal Hindilerden, Gülçin Yeğen, Meliha Nalçacı

**Affiliations:** ^1^Istanbul University, Istanbul Medical Faculty, Department of Internal Medicine, Istanbul, Turkey; ^2^Istanbul University, Istanbul Medical Faculty, Department of Internal Medicine, Division of Hematology, Istanbul, Turkey; ^3^Istanbul University, Istanbul Medical Faculty, Department of Pathology, Istanbul, Turkey

## Abstract

Multiple myeloma is a type of plasma cell disorder and can be seen in different forms. According to current knowledge, it is not a curable disease. Smoldering multiple myeloma (SMM) is an asymptomatic clonal plasma cell disorder and distinguished from monoclonal gammopathy of undetermined significance by a much higher risk of progression to multiple myeloma. We present a 53-year-old female patient who started with SMM which turned into multiple myeloma after four years. Despite 26 cycles of lenalidomide treatment, we performed the second autologous stem transplantation. After 12 years from the diagnosis of the disease, it was transformed into plasma cell leukemia and widespread nodular lesions were seen in the liver. Different presentations could be seen due to malignant plasma cell infiltrations or primary amyloidosis. Liver involvement is one of them and is less common than other organ involvement. We report a case of myeloma presenting with extensive nodular involvement in the liver and misdiagnosed as metastatic disease. It is important because of its rarity and change of the treatment approach.

## 1. Introduction

Different presentation forms and different treatment regimens are available in multiple myeloma. Liver involvement is one of the different presentation forms, and it can mimic metastatic disease.

## 2. Case

A 53-year-old female patient presented with gastrointestinal complaints in June 2006. The general medical examination is unremarkable. She was with no significant medical history. The laboratory findings were within normal limits. A serum protein electrophoresis was performed that demonstrated a monoclonal IgG protein of 1.52 g/dL, and the free kappa/lambda light chain ratio was 12 : 5. There were no bone lesions in bone radiographs or skeletal x-rays. Bone marrow biopsy showed normocellular marrow with 20% plasmositosis. Conventional cytogenetics were normal. So, the patient was diagnosed as IgG kappa smoldering myeloma and had started to follow-up without treatment.

After 4 years, in January 2010, the patient progressed to multiple myeloma with anemia, and a bortezomib-based regimen (bortezomib, cyclophosphamide, and dexamethasone) (CyBorD) was given. After achieving a very good partial response, peripheral stem cells were collected with a filgrastim-alone mobilization regimen. Then, autologous stem cell transplantation was performed with high-dose melphalan (200 mg/m^2^) in December 2010. Maintenance therapy was not given due to the country's reimbursement.

In the fourth year after transplantation, in May 2014, she experienced first nonaggressive relapse and received a lenalidomide and dexamethasone regimen. Anemia and monocytosis occurred during the second year of the lenalidomid treatment. On peripheral blood, the plasma cell count was 2000/mm³. Bone marrow biopsy showed a hypocellular marrow with 30% plasmositosis. At this stage, conventional cytogenetic and FISH studies (*t* (14; 20), *t* (6; 14), *t* (4; 14), *t* (14; 16), *t* (11; 14), 11q13, and 17p13) were evaluated as normal. In June 2016, CyBorD therapy was initiated again due to the complete response with the bortezomib-based regimen previously and relapses more than 5 years after initial treatment, and the patient's performance was not compatible with high-dose combined regimens (VDT-PACE, DICEP).

After achieving a very good partial response again, although she received 26 cycles of lenalidomid, peripheral blood stem cell harvesting was tried with high doses of cyclophosphamide and etoposide plus filgrastim. Stem cells were collected, and transplantation was performed with 140 mg/m^2^ melphalan for the second time, in March 2017. Posttransplant maintenance therapy was not given due to unresponsiveness to previous lenalidomide therapy.

**Figure 1:**
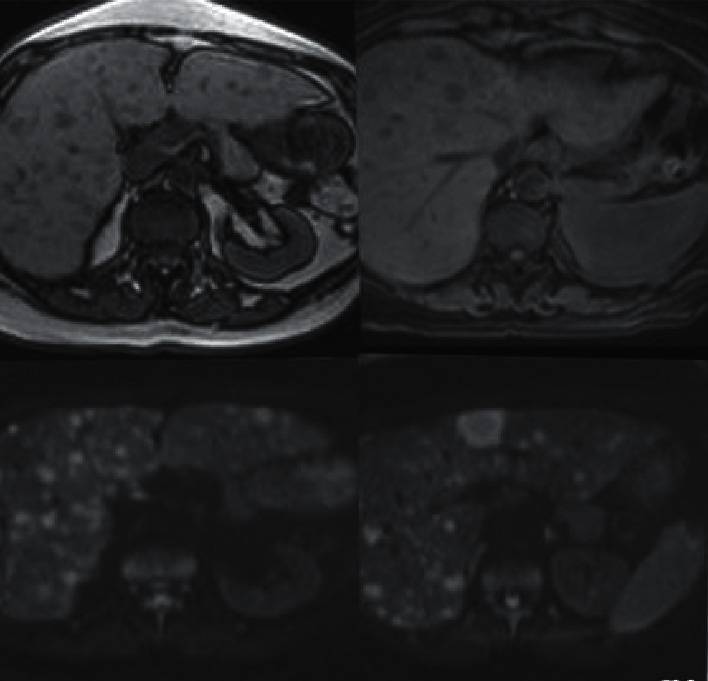
Increased liver size and multiple diffusely distributed lesions which are hyperintense in T2A series and hypointense lesions in T1A series are seen.

**Figure 2:**
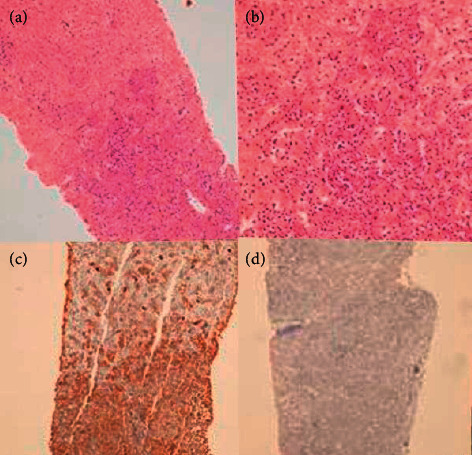
(a, b) Liver paranchyme is infiltrated by plasma cells and full of atypical cells. (c) Kappa (+) neoplastic plasma cells infiltrations are seen. (d) Lambda (−) neoplastic plasma cells infiltrations.

About 1 year after the second transplantation, the patient was admitted to the hospital with right upper quadrant pain over the last two days. On the physical examination, she was pale and had painful hepatomegaly. The complete blood count values were similar with the last one. But, some biochemical tests were abnormal. Aspartate aminotransferase 26 U/L (5–42), alanine aminotransferase 35 U/L (5–42), alkaline phosphatase 122 U/L (35–104), and gama glutamintransferase 234 U/L (5–85) were detected. Tumor markers were evaluated: CA 125 (cancer antigen 125) was 2096 U/ml (<35), and CA 15–3 value was 374 U/ml (<25). There were multiple hypodense lesions on the liver, the largest one being 3 cm length, on the images of the abdomen, Figure 1. Biopsy was taken from the liver lesions and pathological evaluation of the lesion biopsy revealed CD38 (+) Kappa (+) Lambda (−) plasma cell infiltration. There were big patchy grouped atypical cells infiltrations Figure 2. These findings were related with the diagnosis of kappa light chain neoplastic plasma cell infiltration. The patient was dead in the following 2 months, although the chemotherapeutic regimens and supportive cares were given.

## 3. Discussion

Liver involvement is uncommon in the course of multiple myeloma. This involvement is usually due to plasma cell infiltration or concomitant amyloidosis. In one study, Talamo et al. found that gastrointestinal system involvement was present in 24 of 2584 myeloma patients. Liver involvement was seen in 11 of them [1]. However, microscopic plasma cell infiltration is observed more frequently. Thomas et al. reported that, in an autopsy series including 64 patients, 40% had plasma cell infiltration of the liver [2, 3]. Liver involvement may be nodular, sinusoidal, or diffuse or may be in a mixed pattern [4]. Soft tissue involvement may be seen due to decreased expression of adhesion molecules, downregulation of chemokine receptors, downregulation of tetraspanins, increased heparanase-1 expression, angiogenesis, and mutations in alternative or classical nuclear factor-kB pathways [4, 5]. Liver involvement in extramedullary myeloma is found as hypoechoic nodules on the CT scan. Rarely, it may present as hyperechoic nodules and hypervascular lesions [6–8]. It is sometimes confused with metastatic tumors due to the involvement pattern. The fact that the disease is seen in the course of the disease usually plays an important role in revealing the etiology. However, multiple lesions may be seen in the liver in the first diagnosis of the disease, rarely. Definitive diagnosis is made by biopsy from the existing lesions.

Another important feature of this case is that stem cells could be collected after 26 cycles of lenalidomide treatment. It is known in the literature that lenalidomide treatment, especially 4 cycles and above, is a risk factor for mobilization failure [9]. However, this is not a rule, and stem cell harvesting may be attempted in patients who have received 4 cycles and above with lenalidomide therapy.

## 4. Conclusions

Although extramedullary/soft tissue involvement is a rare form of multiple myeloma, it has become more common with the development of imaging techniques. It is not clear whether extramedullary involvement is poor cytogenetic and has negative prognostic features. When the first presentation is with extramedullary involvement, it may cause a delay in diagnosis. New treatment modalities are required, since there is no known treatment method that can provide complete remission, except for allogeneic stem cell transplantation. In selected patients whose treatment options have been exhausted, the autologous transplantation option may be considered once again, despite the number of lenalidomide cycles.

## Data Availability

The data used to support the findings of this study are available from the corresponding author upon request.
